# Cues of High and Low Body Weight Negatively Influence Adults' Perceptions and Ratings in the Hypothetical Adoption Paradigm

**DOI:** 10.1100/tsw.2006.261

**Published:** 2006-12-06

**Authors:** Anthony A. Volk, Vernon l. Quinsey

**Affiliations:** Department of Child and Youth Studies, Brock University, St. Catherines, Ontario and Department of Psychology, Queen's University Kingston, Ontario, Canada

**Keywords:** body weight, parental care, child facial cues, infant facial cues, adoption preference, Canada

## Abstract

Infant and child facial cues influence perceptions and ratings in the Hypothetical Adoption Paradigm as well as actual parental care. A previous study demonstrated that infant and child facial cues of low body weight negatively influenced adults' ratings. The current study sought to replicate and expand on those results by presenting adults with normal faces as well as faces that were digitally altered to display high or low body weight. Cues of abnormal body weight significantly, and negatively, influenced adults ratings of adoption preference, health, and cuteness. Effect sizes were larger for cues of high body weight. Thus, infant and child facial cues of abnormal body weight may represent a relative risk factor to the quality of adult care obtained by children with abnormal body weight.

### INTRODUCTION

Low body weight is associated with a number of serious infant and child health problems[1]. For example, low birth weight infants have much higher levels of morbidity[2] as well as greater risk of physical, neurological, and behavioral developmental deficiencies[3,4]. Another problem associated with low body weight is "failure to thrive"[5]. When infants fail to gain sufficient weight after birth, they are at risk for severe and irreversible physical and mental developmental deficits if the child is unable to gain sufficient weight[6]. Indeed, some pediatric texts regard any weight loss in infants, children, or adolescents as a "highly significant event", possibly signifying serious underlying health problems[7]. Thus, low body weight appears to be a good indicator of several health problems among infants, children, and adolescents.

Compared to low body weight, high body weight in children was originally thought to be associated with fewer immediate health risks[8], but there is mounting evidence that numerous immediate health risks, such as gallstones, hepatitis, hypnoventilation, sleep apneas, and increased intracranial pressure, are associated with childhood obesity[9,10]. The secondary health risk of childhood high body weight is an increased likelihood of being overweight as an adult and a slight increase in adult mortality[10,11,12]. Being overweight as a child also carries a significant mental health risk because overweight children are at greater risk for self-esteem problems and social marginalization[10,13]. Thus, like low body weight, high body weight also appears to be a good predictor of several health problems among children and adolescents. Unlike low body weight, high body weight appears to be a growing risk for Western children[14,15].

Besides these health risks, children with abnormal body weight may also face the risk of receiving poorer parental care. Studies have documented that premature infants, as well as infants and children with health problems, sometimes receive less affection, attention, and/or care from their parents[16,17,18,19,20,21]. Studies of obese children also reveal that childhood obesity is correlated with single, poor, harsh, and/or abusive parents[22]. Poor or reduced parental care can result in physical[23], behavioral, and emotional[24,25,26] problems in children. Infants and children with abnormal body weight may therefore have a higher risk of developing physical, behavioral, and/or emotional problems due to poor or reduced parental care.

A previous study by Volk et al.[27] examined whether facial cues of low body weight significantly influenced adults' ratings in the Hypothetical Adoption Paradigm (HAP). The HAP attempts to measure judgmental processes associated with parental care by asking adults to rate (using a Likert scale) their desire to adopt infants hypothetically based on pictures of the infants' faces. This paradigm has proven successful in the study of a number of different infant and child facial cues (viz., health[27,28,29], resemblance[28,30,31], and age[32]). The paradigm is based on the hypothesis that adults have evolved cognitive mechanisms that allow them to detect important infant and child facial cues and respond to them in ways that would maximize their Darwinian fitness in an ancestral environment (see [27] for more detail). This does not imply that any such mechanisms are entirely innate, morally desirable, or independent of learned experience. Volk et al.'s study of cues of low body weight in the HAP[27] demonstrated that infant and child facial cues of low body weight significantly, and negatively, influenced adults' ratings of adoption preference, health, and cuteness. These results raised an obvious question – how would adults respond to cues of high body weight in the HAP?

### Current Study

The current study sought to address two related questions. First, do infant and child facial cues of high body weight influence adults' perceptions and ratings in the HAP? Second, if adults are influenced by cues of high body weight, is this influence similar to the influence of cues of low body weight?

In the current study, we presented adults with infant and child faces that were unaltered, faces that were digitally altered to simulate low body weight, and faces that were digitally altered to simulate high body weight. To obtain a related measure of desire to provide parental care, we asked adults to rate the cuteness of the faces. Infant facial cues of attractiveness have been found to influence the quality and quantity of parental care[33]. Indeed, infants that were independently rated as less attractive were actually less likely to be safely buckled into grocery carts[34]! Finally, we asked adults to rate the health of the faces in order to get a direct measure of perceived healthiness (both cuteness and health were rated using a Likert scale). We had two main predictions. First, we predicted that adults would give higher adoption preference, health, and cuteness ratings to infants and children whose facial appearance reflected normal body weight than those whose faces had been digitally manipulated to simulate high or low weight. Second, we predicted that adults would respond equally to cues of high and low body weight.

## METHODS

We recruited participants from the Queen's University undergraduate population and from the local community. Undergraduate students from the Psychology 100 Subject Pool received credit towards their course grade, while community members and nonpsychology students received $5 for their participation. While the race of the participants was mixed, non-Caucasian participant data was not included in this study. We also excluded the one participant who answered “Yes” to the screening question: “Do you have significant objections to adoption?” We contacted the community members and nonpsychology students through word of mouth, e-mail, and newspaper advertisements. Most community participants were members of the general Kingston, Ontario community. Participants were excluded if they had participated in the previous low body weight study. We recruited a total of 66 eligible participants. Of these, we excluded three because of a failure to follow instructions. The final sample consisted of 44 women and 19 men. There were 31 undergraduates, 24 women and 7 men (mean age = 19.0, SD = 2.0) and 32 community members, 20 women and 12 men (mean age = 35.9, SD = 12.4). The undergraduates had no children, whereas some community participants did (M = 0.71, SD = 1.2). The undergraduates came from higher SES backgrounds (modal household income of $40–80,000) than the community members (modal household income of $20–40,000).

### Materials

The stimuli consisted of two pictures from each of five different children, one at 18 months of age and the other at 48 months. The children faced the camera in all pictures and the pictures were in color. The stimuli were the same as used in the previous study[27]. All of the children were Caucasian.

Previously, we warped the stimuli to reflect a 10% reduction in apparent facial (and thus body) weight using Gryphon Morph Version 1.5 software[35]. We also performed a second warp to simulate fluctuating asymmetry in the previous study. In the current study, we replaced the fluctuating asymmetry images with images warped to display a 10% increase in apparent facial weight. The 10% increase in body weight was chosen to counter balance experimentally the 10% decrease in body weight used in the previous study[27]. We achieved this digital 10% change in apparent facial weight using two steps. First, we measured the distance between a standard central reference point on the face (the middle of the philtrum) and six other points placed symmetrically along either side of the jaw line starting at the ear lobe and extending to the lower midpoint of the chin. Second, we altered these distances from the external points to the central point by 10%, thereby giving the child an appearance of sunken cheeks and a slightly more protruding chin for low body weight, or the opposite for high body weight. An example of the results of this procedure is presented in Fig. 1. These unaltered and altered images (20 in total) were included with 35 other images used in the previous study (the 35 images were of the same children at different ages). Thus, the total number of images, as well as the number of altered images, remained constant between both the studies.

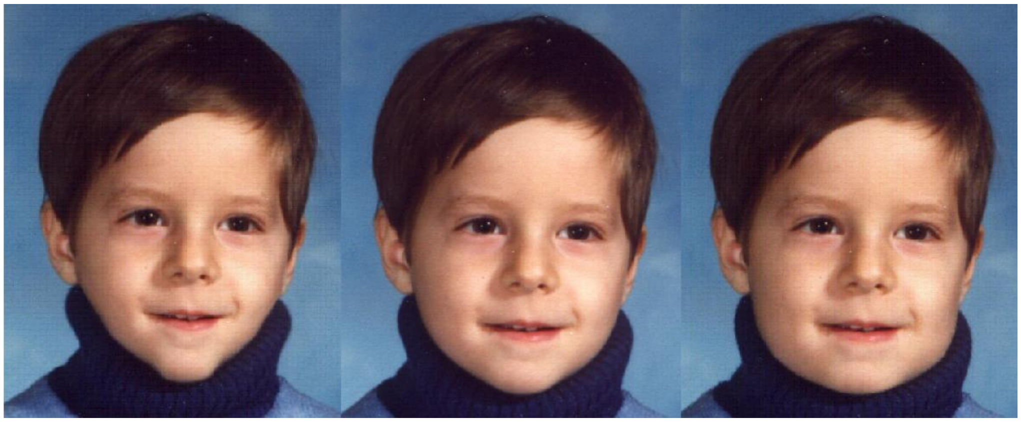

**FIGURE 1**. Example of low, unaltered, and high body weight faces.

As in the low body weight study, the images were presented using Microsoft PowerPoint 2000 software. We generated ten different presentation orders to minimize any order effects. The only restriction for image order selection was that a manipulated picture could not be placed immediately prior to or after its unaltered counterpart. Randomized number tables assigned each participant one of the ten presentation orders. Within each presentation, each image of a child's face was followed by four separate slides on which four separate questions were posed. The order of these questions was kept constant.

### Procedure

The procedure generally matched that of the previous study[27]. Briefly, participants sat by themselves at a desk with a computer where they were presented with the 55 individual faces. A series of four questions followed each stimulus presentation (i.e., after each face). "How willing would you be to adopt the previous child?", "How healthy do you think the previous child is?", "How much do you think the previous child resembles you?", and "How cute do you think the previous child is?" Participants answered these questions using a Likert scale of 1 to 7, where a value of 1 represented the lowest possible score ("very unwilling/unhealthy/no resemblance/not cute"), and a value of 7 represented the highest possible score ("very willing/healthy/high resemblance/very cute"). Our previous work suggests that there is not a significant difference between asking participants to answer these questions individually or asking them answer them simultaneously[28,29]. Following the slide presentation, participants completed a demographic and a personality questionnaire that provided data that were used in a concurrent study. Ratings of resemblance were used to insert a rating judgment that was relatively independent of the other variables in order to reduce method variance in participants' responses.

## RESULTS

Participant ratings were averaged across infant faces to yield average scores for the unaltered faces, the low body weight faces, and the high body weight faces. We performed all of the analyses using SPSS 11.0, with a base $\alpha = 0.05$ for all analyses. We used a repeated measures MANOVA to evaluate our predictions that participants would give significantly lower ratings of adoption preference, cuteness, and health to the high and low body weight faces as compared to the normal faces, while simultaneously examining the roles of participant Group and Sex. We also evaluated whether there were significant differences between the ratings of the high and low body weight faces. The variables did not significantly violate univariate statistical assumptions.

### Stimuli Weight Analysis

The average ratings of adoption preference, cuteness, and health for the low, normal, and high body weight faces are presented in Table 1. An ANOVA revealed that there were no significant differences between the average adoption preference, cuteness, and health ratings of the low and unaltered body weight in the current study and those obtained in the previous study[27]. The values for the ratings in the current study were analyzed using a $2 \times 2 \times 3 \times 3$ Repeated Measures MANOVA using Pillai's Trace for the multivariate analyses (a conservative test[36]). There were significant between group effects for Sex ($F(3,57) = 3.60$, $p < 0.05$, partial $\eta 2 = 0.16$) and Group ($F(3,57) = 6.95$, $p < 0.001$, partial $\eta 2 = 0.27$), with women and community members giving higher average ratings. There was a significant within-group effect for Stimuli Weight ($F(6,54) = 2.35$, $p < 0.05$, partial $\eta 2 = 0.21$). Due to violations of sphericity, the univariate results degrees of freedom were adjusted using the conservative Greenhouse-Geisser correction[36]. There were significant differences in the ratings of adoption preference ($F(2,60) = 6.54$, $p < 0.01$, partial $\eta 2 = 0.10$), cuteness ($F(2,60) = 4.24$, $p < 0.05$, partial $\eta 2 = 0.07$), and health ($F(2,60) =$

**TABLE 1**
**Means and SD for High, Normal, and Low Body Weight Faces (n = 63)**

| | Low | Normal | High |
|---|---|---|---|
| Adoption | 4.26 (1.21) | 4.36 (1.17) | 4.13 (1.26) |
| Cuteness | 4.72 (1.09) | 4.85 (1.05) | 4.59 (1.14) |
| Health | 4.52 (0.98) | 4.76 (0.91) | 4.58 (1.03) |

6.20, $p < 0.01$, partial η2 = 0.10). The average low, normal, and high body weight facial cue ratings for adoption preference, cuteness, and health are presented in Fig. 2. Tests of within-subject polynomial contrasts revealed larger effect sizes (i.e., a better fit) with quadratic analyses as compared to linear analyses. Repeated contrast analyses revealed that the ratings for high body weight were significantly different from the ratings for normal body weight for all three dependent measures. The same analyses revealed that the ratings of low body weight differed from the ratings for normal body weight for adoption preference and health, but not cuteness. Simple contrast analyses tested between the ratings of the low and high body weight faces, and found small, but significant, differences. The high body weight faces received significantly lower ratings of adoption preference ($F(1,61) = 4.05$, $p < 0.05$, partial η2 = 0.07) and cuteness ($F(1,61) = 4.62$, $p < 0.05$, partial η2 = 0.07). There was no significant difference between the ratings of health for the low and high body weight faces.

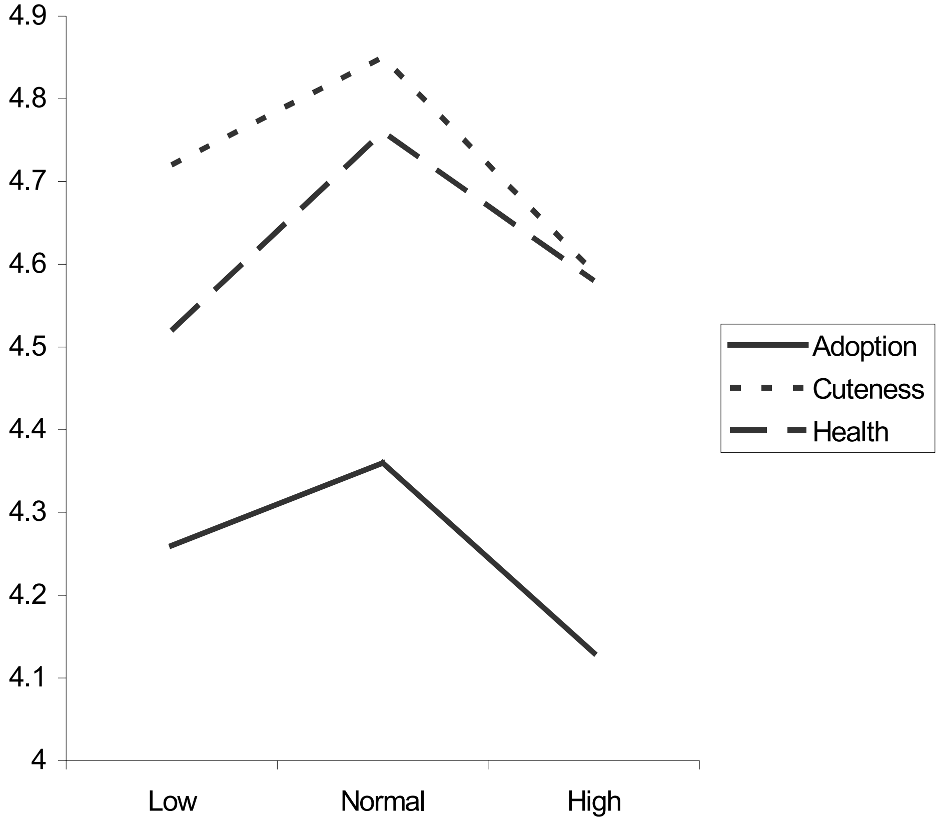

**FIGURE 2**. Average ratings of adoption preference, cuteness, and health for the low, normal, and high body weight faces.

### Within-Stimuli Average Correlations

The average within-stimuli correlations were calculated by averaging the correlations across stimuli (see[28] for further details). The results are presented in Table 2. As can be seen, all of the correlations (except for resemblance-health in unaltered faces) were significant. While there appears to be a trend towards higher correlations between adoption preference and health for the altered faces, there were no significant differences between the low, normal, and high body weight correlations when the correlations were simultaneously analyzed at a multivariate level.

**TABLE 2**
**Within-Stimuli Average Correlations**

| | | Cuteness | Health | Resemblance |
|---|---|---|---|---|
| Adoption preference | Low | 0.700* | 0.458* | 0.296* |
| | Unaltered | 0.643* | 0.339* | 0.225* |
| | High | 0.684* | 0.399* | 0.295* |
| Cuteness | Low | — | 0.513* | 0.304* |
| | Unaltered | — | 0.468* | 0.276* |
| | High | — | 0.518* | 0.326* |
| Health | Low | — | — | 0.224* |
| | Unaltered | — | — | 0.120 |
| | High | — | — | 0.170* |

* = $p < 0.005$ (Dunn adjustment of the original $\alpha = 0.05$).

### DISCUSSION

As predicted, participants gave significantly lower ratings of adoption preference, cuteness, and health to the faces that were digitally altered to display cues of high and low body weight. The lower health ratings for the faces with altered weight suggest that the digital manipulations were successful in simulating cues of poorer health, while the lower ratings for adoption preference and cuteness suggest that facial cues of high and low body weight in infants and children significantly influenced cognitive processes associated with parental care. Our results agree with previous findings[27], and support the conclusion that infant and child facial cues of body weight can significantly influence adults' perceptions and ratings in the HAP. As in previous studies of cues of health and the HAP, there were no significant differences among the correlations between the variables. This suggests that the observed differences are likely due to absolute differences in the variables, rather than different variable relationships among the manipulations. The correlations of resemblance with health and cuteness were small, suggesting that the use of resemblance as a distracter variable to minimize method variance was appropriate.

Women gave more positive ratings to the child faces than did men. This result agrees with both previous studies that used the HAP[27,30], as well as women's generally greater interest in, and care of, children[37]. Our results further strengthen the findings that adult sex differences exist with regards to the perception of and reaction to infant and child faces. We also found that community members gave higher average ratings than did undergraduates. Results from a concurrent study of parenting and childcare experience, as well as adults' personality traits, suggest that differences between parenting experience, number of children, and personality traits underlie group rating differences in the HAP (Volk and Quinsey, in preparation).

In contrast to our second prediction, participants did not respond equally to the different manipulations. Participants gave significantly lower ratings of adoption preference and cuteness to the high body weight faces. Participants did not differ in their health ratings for the two different manipulations. These results suggest that compared to cues of low body weight, cues of high body weight factors may present adults with extra negative cues that are associated with cuteness, but not health. A second possibility is that the Western bias of associating high body weight with unattractiveness[38] may extend to child and infant faces. A related third possibility is that cues of high body weight may not only suggest to adults immediate negative correlates, but also long-term negative correlates. For example, adults may believe that high body weight children are at risk for being high body weight adults, and thus at risk for continued health risks, decreased attractiveness, and social marginalization. Any or all of these explanations could account for the significant differences in adoption preference and cuteness, but not health.

In any case, the current results make an important methodological contribution regarding the validity of the HAP; that adults' responses are influenced not only by the presence of a manipulation, but by the type of the manipulation that is performed. For example, in the current study, a high body weight manipulation produced a greater effect than a low body weight manipulation. In the previous study, cues of low body weight produced a significant effect, while cues of fluctuating asymmetry did not. In the study of fetal alcohol syndrome, healthy faces altered to display fetal alcohol syndrome were rated more negatively, while faces with fetal alcohol syndrome that were altered to appear "normalized" were rated more positively. Anecdotally, in the present study, adults reported seeing many more infant faces than were actually used in the study (i.e., they didn't recognize the same child at different ages and/or body weights). Taken as a whole, these results suggest that adults' responses to facial cues in the HAP are not primarily influenced by the repeated presentation of faces or by the mere presence of a digital manipulation. Rather, adults appear to be responding primarily to the specific type of manipulation that is performed.

#### Limitations

The clinical significance of the current effect sizes should be interpreted cautiously for several reasons. First, there is no direct translation of the results from the HAP to actual differences in parental care. Second, as in the previous study, the means for the groups were all above the median of the Likert scales use to measure health, cuteness, and adoption preference. Third, the small effect sizes found in this study may be a function of the conservative degree of digital manipulation. A 10% reduction or increase in body weight is typically not immediately life threatening in either infants or children[7], and such changes cause relatively subtle changes in facial appearance. Thus, the small effect sizes demonstrated in the current study most likely represent the lower range of possible adult reactions to cues of abnormal body weight in children's faces. The employment of more dramatic weight changes, a more thorough manipulation of body weight across the entire body, and/or the employment of a forced-choice paradigm might all yield larger effect sizes. We did not use larger manipulations because we wanted a conservative test of facial cues and we wanted to avoid manipulations that looked unnatural. Fourth, it is worth noting that pure variance explanations of effect size can underestimate the actual effect of cumulative events[39,40]. The differences found in this study apply to a "one-shot" static interaction. Actual care of a child or infant involves many repeated interactions, during which the differences observed in this study could be summed over time to produce a larger effect. Finally, the five children used as stimuli may not be entirely representative of children in general.

### CONCLUSIONS

The digital manipulations of cues of high and low body weight in the faces of infants and children proved successful in creating the appearance of relatively diminished health using the HAP. Compared to unaltered faces, digitally altered high and low body weight faces received significantly lower ratings of adoption preference, cuteness, and health. High body weight faces received significantly lower adoption preference and cuteness ratings, suggesting that adults view cues of high body weight even more negatively than they do cues of low body weight. Women were generally more positive in their ratings than men, and members of the community gave higher average ratings than undergraduates. The differences between the weight conditions observed in this study were not due to different correlational relationships among the rating variables.

Our results confirm and expand on those of our previous study of low body weight and suggest that facial cues of abnormal body weight in infants and children may represent a relative risk factor. In particular, facial cues of abnormal body weight appear to influence perceptions and ratings negatively related to parental care in adults, potentially placing infants and children with such cues at risk for diminished quality and/or quantity of parental care. Given the significant number of children with abnormal weight, it is important for doctors, clinicians, and parents to recognize that there may be implicit negative adult biases towards abnormal weight children. Those biases have the potential to influence negatively the quality and/or quantity of parental care that a child may receive[33,34]. Such biases may be counteracted by both raising the weight of the child and/or by providing parents and caregivers of abnormal body weight children with the knowledge and resources required to overcome any biases.

A further consideration is that an increasing number of adoption agencies are employing photo albums of infant and child faces, both on and off the Internet, to promote the adoption of children in their care. Such agencies should be aware of the potentially negative influences associated with photographs taken of children who are of abnormal body weight. Attempts to stabilize any medical or weight conditions prior to taking the photographs may improve the chances of a particular child being successfully adopted. At the very least, if agencies are aware of the importance of different facial cues, they can act in ways that best demonstrate the positive traits of each child, thereby maximizing chances for successful adoption.
